# Selecting against accidental RNA interactions

**DOI:** 10.7554/eLife.20686

**Published:** 2016-09-20

**Authors:** Meredith Corley, Alain Laederach

**Affiliations:** Department of Biology, University of North Carolina, Chapel Hill, United Statesmcorley@email.unc.edu; Department of Biology, University of North Carolina, Chapel Hill, United Statesalain@unc.edu

**Keywords:** ncRNA, Archaea, Bacteria, gene expression, bioinformatics, *E. coli*, Other

## Abstract

Random base-pairing interactions between messenger RNAs and noncoding RNAs can reduce translation efficiency.

**Related research article** Umu SU, Poole AM, Dobson RCJ, Gardner PP. 2016. Avoidance of stochastic RNA interactions can be harnessed to control protein expression levels in bacteria and archaea. *eLife* **5**:e13479. doi: 10.7554/eLife.13479

Translation is the process by which the genetic information in a molecule of messenger RNA (mRNA) produces a protein, and the rate at which protein is produced from a given mRNA molecule is called the translation efficiency. This number is different for different mRNA molecules (Maier et al., 2009; Guo et al., 2008), which is why researchers are trying to determine which features of these molecules affect their translation efficiency (Tuller et al., 2010; Ferreira et al., 2013; Kozak, 2005; Gingold and Pilpel, 2011).

Now, in eLife, Paul Gardner of the University of Canterbury and colleagues – including Sinan Umu (as first author), Anthony Poole and Renwick Dobson – report that the translation efficiency in bacteria and archaea is influenced by a phenomenon called "avoidance" (Umu et al., 2016). Avoidance is the degree to which an mRNA molecule avoids random interactions with noncoding RNA molecules in the cell. Noncoding RNAs, as their name suggests, do not code for proteins, but they still make up a majority of the RNA in any given cell. Indeed, the researchers show that the levels of noncoding RNAs in bacterial cells are two orders of magnitude greater than the levels of mRNAs.

To estimate the probability of random base-pairing interactions taking place between mRNAs and noncoding RNAs, consider a five-base region in a single mRNA. This region can have any one of a possible 4^∧^5=1024 sequences. If the total number of bases from all the noncoding RNAs in the cell is S, then the number of noncoding RNAs in the cell that have a perfectly complementary five-base region is approximately S/1024. Umu et al. studied 325 noncoding RNAs so, assuming an average length of 200 bases for these, we have S ≈ 325*200 ≈ 65000. Therefore, on average, the number of these noncoding RNAs that have a five-base region that is perfectly complementary to the five-base region in the mRNA will be 65000/1024 ≈ 63. Given the number of mRNAs and noncoding RNAs that are found in cells, random interactions between the two are inevitable. However, if we find that a given mRNA has base-pairing interactions with fewer noncoding RNAs than expected, then this is avoidance (Figure 1A).

**Figure 1.**
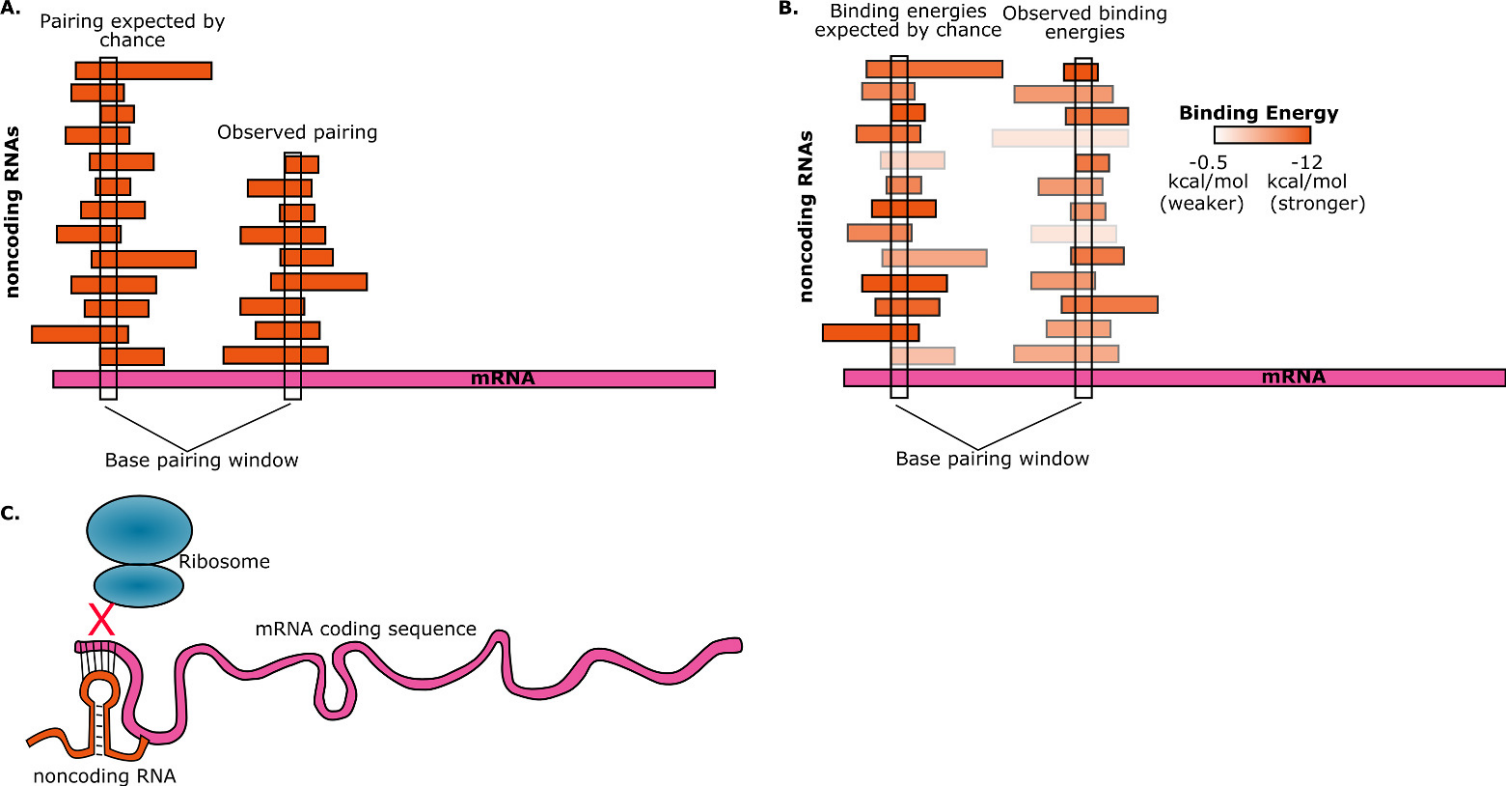
Random interactions between mRNAs and noncoding RNAs. (**A**) A given region of mRNA (pink) should undergo random base-pairing interactions with a certain number of noncoding RNAs (orange). However, a phenomenon called "avoidance" results in the number of observed pairings (right) being fewer than the number expected by chance (left). This simplified picture does not allow for the influence of binding energies and other effects. (**B**) The ability of RNA molecules to fold into complex structures means that mRNA-noncoding RNA interactions can have a range of binding energies (see color bar). "Avoidance" results in the observed binding energies (right) being, on average, weaker than the binding energies expected by chance (left). Umu et al. report a significant difference in binding energy distributions in 73% of bacterial and archeal species. (**C**) If a random base-pairing interaction results in a noncoding RNA pairing with part or all of a start codon in an mRNA, the ribosome will not be able to translate the mRNA.

Realistically, RNA binding interactions are governed by thermodynamics and do not always follow strict pairing rules. Furthermore, RNA molecules can pair with themselves in intra-molecular interactions and must “unfold” a given region in order to pair with another molecule. Thus it is important to quantify mRNA-noncoding RNA interactions with net "binding energy" calculations. The binding energy quantifies the thermodynamics of RNA base-pairing, with low binding energies indicating very stable interactions. To explore the phenomenon of avoidance, Umu et al. used a computational RNA interaction model to estimate the binding energies for interactions between mRNAs and noncoding RNAs. The RNAs include a core set of 114 mRNAs that are well conserved across bacteria (including 40 that are also conserved across archaea) and 325 noncoding RNAs from six families of RNA that are also well conserved across bacterial and archaeal species.

They found that, on average, the interactions between the core noncoding RNAs and mRNAs were weaker than the interactions between the noncoding RNAs and a control set. In other words, they found that the average mRNA "avoids" interactions due to less stable pairing with noncoding RNAs (Figure 1B). This trend holds true for over 70% of the bacteria and archaea that they tested. There are, of course, noncoding RNAs whose primary function is to bind to mRNAs, but these were excluded from the study. Instead, the goal was to observe selection against *accidental* interactions between mRNAs and the large and diverse set of noncoding RNAs that are resident in the cell.

Umu et al. hypothesize that avoidance is due to the negative effect that the interactions between mRNAs and noncoding RNA could have on translation efficiency: for example, if a noncoding RNA pairs with a start codon in an mRNA, it will prevent translation from taking place because the ribosome will not be able to bind to that mRNA (Figure 1C). To test this hypothesis, the researchers designed and synthesized a set of mRNAs with sequences that have high levels of avoidance, and a set of mRNAs with low levels of avoidance. When they measured the translation efficiency for both sets of mRNAs, they found that it was much higher for the highly-avoidant set.

Umu et al. also synthesized different sets of mRNAs to explore two other factors that are thought to influence translation efficiency: codon bias and the intra-mRNA folding energy (Kudla et al., 2009; Tuller et al., 2010). Both factors did cause some variation in the production of protein, but avoidance was responsible for the most variation. They also found the same correlation with avoidance when they studied previously published measurements of bacterial translation efficiency. This suggests that the ability of an mRNA to avoid interactions with noncoding RNAs is a hitherto unknown, yet important factor affecting translation efficiency.

One notable aspect of this study is that it relied almost entirely on publicly available data sets. This underscores the importance of open data for exploring basic biological questions that apply to many different organisms. Using this data, which no single lab could have collected alone, Umu et al. have shown that mRNA sequences are optimized to minimize interactions with noncoding RNAs and have demonstrated why such avoidance is so desirable. And the need to avoid spurious interactions is not unique to RNA: the emergence of complex life depends on optimizing molecular interactions that lead to reproduction in the midst of molecular chaos. Although networks of highly specific molecular interactions are a hallmark of evolution, in many cases it is just as important to avoid accidental interactions.
